# Prognostic impact of early disease progression and early mortality in patients with multiple myeloma: a real-world cohort study

**DOI:** 10.3389/fonc.2026.1812031

**Published:** 2026-05-26

**Authors:** Nada Vlaisavljevic, Ivana Milosevic, Borivoj Sekulic, Jelena Nikolic, Velimir Tomic, Aleksandar Savic

**Affiliations:** 1 Faculty of Medicine, University of Novi Sad, Novi Sad, Serbia; 2 Clinic of Haematology, University Clinical Center of Vojvodina, Novi Sad, Serbia

**Keywords:** multiple myeloma, early disease progression, POD18, early mortality, dynamic prognostic markers, overall survival

## Abstract

**Introduction:**

Multiple myeloma (MM) is a clinically heterogeneous malignancy in which outcomes remain difficult to predict using static baseline risk models. Increasing attention has shifted toward dynamic prognostic markers, including early disease progression and early mortality; however, their relative prognostic value—particularly the optimal definition of early progression—and their integration with patient-related factors remain insufficiently defined in real-world populations.

**Methods:**

We retrospectively analysed 207 newly diagnosed MM patients treated between 2018 and 2023. Early disease progression was defined as progression within 18 (POD18) and 24 months (POD24) from treatment initiation. Early mortality was defined as death within six months of diagnosis. Overall survival (OS) and progression-free survival (PFS) were analysed using Kaplan–Meier estimates and Cox regression models, including time-dependent analyses.

**Results:**

During a median follow-up of 60 months, POD18 occurred in 44.0% and POD24 in 51.2% of patients. POD18 was strongly associated with inferior OS (HR 9.38; 95% CI 5.98–14.70; p < 0.0001) and demonstrated superior prognostic discrimination compared with POD24 (C-index 0.742 vs. 0.719). In multivariable analysis, POD18 remained the strongest independent predictor of OS, alongside comorbidity burden and performance status. Early mortality occurred in 17.9% of patients and was independently associated with advanced disease stage. Time-dependent modelling showed that the prognostic impact of baseline staging decreased over time, supporting the dynamic nature of risk in MM.

**Conclusion:**

POD18 is a robust and clinically informative dynamic prognostic marker that outperforms POD24 in predicting survival in real-world patients with MM. Integration of early disease kinetics with baseline disease burden and comorbidity burden provides a pragmatic framework for dynamic risk stratification, particularly in settings with limited access to comprehensive molecular profiling.

## 1 Introduction

Multiple myeloma (MM) is a biologically and clinically heterogeneous haematological malignancy in which patient outcomes remain highly variable despite substantial therapeutic advances. The introduction of proteasome inhibitors, immunomodulatory drugs, monoclonal antibodies, and the widespread use of autologous stem cell transplantation (ASCT) has significantly improved response rates and survival over the past two decades (1–3). However, MM remains largely incurable, and accurate prediction of disease course in individual patients continues to be challenging.

Current risk stratification strategies rely predominantly on static baseline models, including the International Staging System (ISS) and cytogenetic profiling. While these approaches provide important prognostic information at diagnosis, they do not fully capture the dynamic nature of disease evolution, treatment response, and clinical heterogeneity observed in routine clinical practice (4, 5). Consequently, patients with apparently similar baseline characteristics may experience markedly divergent clinical trajectories, highlighting the limitations of static risk assessment.

In recent years, increasing attention has been directed toward dynamic prognostic markers that reflect disease behaviour over time. Early disease progression has emerged as a particularly strong predictor of adverse outcomes in MM. Several studies have demonstrated that progression within the first 12–24 months following initiation of therapy or ASCT is associated with significantly inferior overall survival, independent of baseline risk factors (6–9). Among these, progression within 18 months (POD18) has been proposed as a sensitive indicator of aggressive disease biology and early treatment failure. However, the optimal time threshold for defining clinically meaningful early progression, and the relative prognostic performance of POD18 compared with the more widely used POD24, remain insufficiently defined, particularly in real-world settings.

In parallel, early mortality represents a critical but relatively underexplored component of MM outcomes. Death occurring within the first months after diagnosis is frequently driven by advanced disease burden, treatment-related toxicity, comorbidities, and infectious complications (10–12). Importantly, early mortality and early disease progression may reflect complementary manifestations of aggressive disease biology, encompassing both tumour burden at presentation and early resistance to therapy.

Despite growing interest in dynamic risk assessment, most available evidence is derived from selected clinical trial populations and focuses predominantly on disease-related factors. In contrast, real-world patients are typically older, have a higher burden of comorbidities, and often present with more advanced disease, all of which may substantially influence both early outcomes and long-term survival. Furthermore, comprehensive cytogenetic profiling is not consistently available in routine practice, limiting the applicability of biology-driven risk models.

In this context, there is a need for pragmatic and integrative approaches that combine baseline disease burden, dynamic disease kinetics, and patient-related vulnerability to better reflect real-world clinical complexity. Outcome-based markers such as early disease progression may serve as accessible surrogates of aggressive disease behaviour, particularly in settings where molecular data are incomplete.

Therefore, in this real-world cohort study of newly diagnosed MM patients, we aimed to (i) evaluate the prognostic significance of early disease progression at 18 and 24 months, (ii) compare the discriminative performance of POD18 and POD24, (iii) assess relapse patterns following ASCT, and (iv) identify predictors of early mortality. By integrating disease burden, early disease kinetics, and comorbidity burden within a single analytical framework, this study seeks to provide a clinically relevant model of dynamic risk stratification applicable to routine practice.

## 2 Materials and methods

### 2.1 Study design and patients

This study included 207 patients diagnosed with multiple myeloma (MM) who received first-line therapy at the University Clinical Centre of Vojvodina between 2018 and 2023 in a retrospective cohort study. The inclusion criteria were a confirmed diagnosis of MM according to the International Myeloma Working Group (IMWG) criteria and available clinical follow-up data after the initiation of treatment (1).

Demographic, clinical, and laboratory data were collected from medical records at diagnosis. All patients received standard first-line antimyeloma therapy according to institutional protocols and treatment availability at the time of diagnosis. First-line treatment regimens included combinations of proteasome inhibitors, immunomodulatory drugs, cyclophosphamide, melphalan, and corticosteroids: bortezomib–cyclophosphamide–dexamethasone (VCD), bortezomib–thalidomide–dexamethasone (VTD), bortezomib–dexamethasone (VD), cyclophosphamide–thalidomide–dexamethasone (CTD), melphalan–prednisone–thalidomide (MPT), melphalan–prednisone–bortezomib (MPV), and melphalan–prednisone (MP). Regimen selection reflected real-world clinical practice and frailty-driven treatment decisions (13).

Autologous stem cell transplantation (ASCT) was performed according to institutional practice and patient eligibility and remains a standard consolidation option for eligible patients in the era of novel agents (6, 7). Disease response, progression, and relapse were assessed using IMWG criteria (2).

### 2.2 Variables and definitions

Baseline variables included age, sex, International Staging System (ISS), Eastern Cooperative Oncology Group performance status (ECOG PS), Charlson Comorbidity Index (CCI), and laboratory parameters (haemoglobin, serum albumin, beta-2 microglobulin, lactate dehydrogenase, renal function, serum calcium, and bone marrow plasma cell infiltration). The selection of these variables followed standard clinical practice and published recommendations (3, 14).

#### 2.2.1 CRAB and SLiM criteria

CRAB and SLiM criteria were assessed at baseline according to IMWG diagnostic definitions. CRAB features included hypercalcaemia (serum calcium >2.75 mmol/L), renal impairment (serum creatinine >177 μmol/L), anaemia (haemoglobin <100 g/L), and bone disease.

Bone disease was assessed using conventional skeletal radiography of the axial and appendicular skeleton (plain radiographs of flat bones), as this represented the standard imaging modality during the study period. Low-dose whole-body computed tomography (LDCT), magnetic resonance imaging (MRI), and positron emission tomography–computed tomography (PET-CT) were not systematically available for all patients; therefore, osteolytic lesions were evaluated uniformly using conventional radiography across the entire cohort.

SLiM criteria included bone marrow plasma cell infiltration ≥60% and an involved/uninvolved serum free light chain (FLC) ratio ≥100, in accordance with IMWG recommendations (2).

#### 2.2.2 Early disease progression and mortality definitions

Early disease progression was evaluated at predefined time points. Disease progression at 18 months (POD18) and 24 months (POD24) from initiation of first-line therapy was analysed in the entire cohort. In patients undergoing ASCT, disease progression after transplantation was assessed at 6, 12, and 24 months (aPD6, aPD12, and aPD24).

Early mortality was defined as death occurring within six months of diagnosis.

Overall survival (OS) was defined as the time from diagnosis to death from any cause or last follow-up. Progression-free survival (PFS) was defined as the time from diagnosis to documented disease progression or death, whichever occurred first.

### 2.3 Statistical analysis

Patient characteristics are summarised as frequencies and percentages for categorical variables and as means with standard deviations or medians with interquartile ranges (IQR) for continuous variables, as appropriate. Survival curves were estimated using the Kaplan–Meier method and compared using the log-rank test.

Univariate Cox proportional hazards regression was performed to identify variables associated with overall survival (OS) and progression-free survival (PFS). Variables with p < 0.10 in univariate analysis were included in multivariable Cox regression models using a forced-entry approach. Multicollinearity among predictors was assessed using variance inflation factors (VIF) and tolerance values. No significant multicollinearity was detected (all VIF values < 5 and tolerance > 0.2), indicating that the included variables were suitable for multivariable modelling. In addition, the number of variables included in multivariable models was limited relative to the number of events to minimise the risk of overfitting, ensuring an adequate events-per-variable ratio.

The proportional hazards assumption was formally assessed using Schoenfeld residuals. Violation of the proportional hazards assumption was observed for PFS. To address this, extended Cox regression models incorporating time-dependent covariates were applied, allowing hazard ratios to vary over time. This approach improves model validity in the presence of non-proportional hazards and provides more accurate estimates of the association between predictors and PFS.

Cox regression results are presented as regression coefficients (β), standard errors (SE), hazard ratios (HR), 95% confidence intervals (CI), and p-values. Prognostic discrimination of the survival models was assessed using Harrell’s concordance index (C-index).

Predictors of early mortality at 6 months were evaluated using logistic regression analysis, given the binary nature of the outcome, with variables significant in univariate analysis included in multivariable models.

All statistical tests were two-sided, and a p-value < 0.05 was considered statistically significant. Statistical analyses were performed using MedCalc^®^ Statistical Software (version 23.4.5) and R software (version 4.6.0).

## 3 Results

### 3.1 Patient characteristics

A total of 207 patients with newly diagnosed multiple myeloma were included in the analysis. The cohort was characterised by advanced disease at diagnosis and a high burden of comorbidities. The mean age was 65.3 ± 10.0 years, with a slight male predominance (54.1%). According to the International Staging System (ISS), 49.8% of patients were classified as stage III. Impaired performance status (ECOG PS > 2) was observed in 29.5% of patients.

A substantial comorbidity burden was present, with 78.1% of patients having a Charlson Comorbidity Index (CCI) ≥ 4. Cytogenetic data were unavailable or non-informative in 83.6% of patients. Baseline disease characteristics are summarised in **Table 1**.

**Table 1:** Baseline demographic, clinical, and laboratory characteristics (N=207).

Variable	Overall (N=207)
Age,years	65.3 ± 10.0
Gender	
Male	112 (54.1%)
Female	95 (45.9%)
ISS stage	
1	37 (17.9%)
2	67 (32.4%)
3	103 (49.8%)
ECOG PS	
≤2	146 (70.5%)
>2	61 (29.5%)
Total protein (g/L)	89.3 ± 23.2
Albumin (g/L)	34.0 ± 8.3
β2-microglobulin (mg/L)	5.1 (1.11–46.37)
LDH (U/L)	185 (79–3370)
Cytogenetic risk (FISH)	
0	173 (83.6%)
1	11 (5.8%)
2	19 (9.2%)
3	3 (1.4%)
Charlson Comorbidity Index (CCI)	
1-3	43 (20.77%)
≥ 4	164 (79.23%)

Values are presented as mean ± standard deviation, median (interquartile range), or number (%), as appropriate. ISS, International Staging System; ECOG PS, Eastern Cooperative Oncology Group performance status; LDH, lactate dehydrogenase; FISH, fluorescence *in situ* hybridization; CCI, Charlson Comorbidity Index.

Cytogenetic risk categories: 0, unavailable or non-informative; 1, standard risk; 2, high risk; 3, double-hit abnormalities.

CRAB and SLiM features indicated a high disease burden, with frequent anaemia, renal impairment, and osteolytic bone disease, as detailed in **Table 2**.

**Table 2:** CRAB and SLiM criteria at diagnosis of the study cohort (N=207).

Variable	Overall (N=207)
Ca (mmol/L)	2.66 (2.24-3.52)
Ca > 2.75mmol/L	67 (32.40%)
creatinine (μmol/L)	112.00 (45.00-1222.00)
creatinine >177 μmol/L	73 (35.30%)
haemoglobin (g/L)	94.00 (52.00-164.00)
haemoglobin<100g/L	118 (57%)
Involved/uninvolved serum free light chain ratio	148.07 (0.01-890.41)
Involved/uninvolved serum free light chain ratio FLC>100	106 (51.20%)
Bone marrow plasma cell infiltration (%)	60.00 (10.00 - 95.00)
>60% bone marrow plasma cells	114 (55.10%)
Osteolytic lesions >10%	132 (63.80%)

Values are presented as median (range), or number (%), as appropriate. Ca, calcium; FLC, free light chain.

First-line treatment predominantly consisted of bortezomib-based regimens, reflecting real-world clinical practice, as detailed in **Table 3**. Autologous stem cell transplantation (ASCT) was performed in 13.5% of patients.

**Table 3:** Distribution of first-line treatment regimens in the study cohort (N = 207).

Treatment regimen	VCD	VTD	VD	CTD	MPT	MPV	MP	Total
N	76	4	20	59	29	15	4	207
%	36.7	1.9	9.7	28.5	14.0	7.2	1.9	100.0

VCD, bortezomib–cyclophosphamide–dexamethasone; VTD, bortezomib–thalidomide–dexamethasone; VD, bortezomib–dexamethasone; CTD, cyclophosphamide–thalidomide–dexamethasone; MPT, melphalan–prednisone–thalidomide; MPV, melphalan–prednisone–bortezomib; MP, melphalan–prednisone.

### 3.2 Response to first-line therapy

Treatment response was evaluable in all patients. A complete response (CR) was achieved in 20.3% of patients, very good partial response (VGPR) in 30.0%, and partial response (PR) in 26.6%. Overall, a deep response (≥VGPR) was achieved in 50.3% of patients. In contrast, 16.4% of patients exhibited primary refractory disease or died early during treatment (**Table 4**).

**Table 4:** Response to first-line therapy in the study cohort (N=207).

Response	N	%
CR	42	20.3
VGPR	62	30.0
PR	55	26.6
SD	14	6.8
PD	16	7.7
ED	18	8.7
Total	207	100.0

CR, Complete Response; VGPR, Very Good Partial Response; PR, Partial Response; SD, Stable Disease; PD, Progressive Disease; ED, Early Death.

### 3.3 Early mortality

Early mortality within six months of diagnosis occurred in 17.9% of patients, indicating a substantial burden of early adverse outcomes. A proportion of deaths occurred during first-line therapy and were primarily related to disease progression (**Table 5**).

**Table 5:** Early outcomes and disease progression in the study cohort (N=207).

Variable	N	%
Early mortality at six months		
No	170	82.10
Yes	37	17.90
Progression of disease at 18 months (POD18)		
No	116	56.00
Yes	91	44.00
Progression of disease at 24 months (POD24)		
No	101	48.8
Yes	106	51.2
Relapse		
No relapse	66	31.9
1^st^	121	58.40
2^nd^	16	7.70
≥3^rd^	4	1.90

Values are presented as number (%) unless otherwise indicated.

N, number of patients; %, percentage; POD18, progression of disease within 18 months; POD24, progression of disease within 24 months.

### 3.4 Causes of early mortality

To further characterise early mortality, the immediate causes of death were analysed. Among patients who died during induction therapy due to disease progression, the leading causes were acute renal failure associated with malignant hypercalcaemia (2.9% of the total cohort), sepsis with renal failure (1.9%), and cardiovascular complications, including decompensated heart failure (1.9%) and thromboembolic events (1.4%). A single case of intracranial haemorrhage was observed (0.5%).

Given that the study period encompassed the COVID-19 pandemic, infection-related mortality was analysed separately. COVID-19–related mortality occurred in 2.9% of patients, while non–COVID-19 pneumonia accounted for 6.3% of deaths.

### 3.5 Relapse patterns

During follow-up, disease relapse occurred in 68.1% of patients, while 31.9% remained relapse-free. The first relapse was documented in 58.5% of the cohort, the second in 7.7%, and three or more relapses in 1.9% (**Table 5**).

### 3.6 Survival outcomes

After a median follow-up of 60 months, the median overall survival (OS) was 52 months (95% CI: 40–62), and the median progression-free survival (PFS) was 24 months (95% CI: 18–30). These outcomes reflect the advanced disease profile and high comorbidity burden of the cohort.

### 3.7 Early disease progression (POD18 and POD24)

Disease progression within 18 months (POD18) occurred in 44.0% of patients and was strongly associated with inferior overall survival (HR 9.38; 95% CI: 5.98–14.70; p < 0.0001). In contrast, progression within 24 months (POD24) was observed in 51.2% of patients and showed a weaker association with mortality (HR 6.64; 95% CI: 4.35–10.12; p < 0.0001).

**Figure 1:**
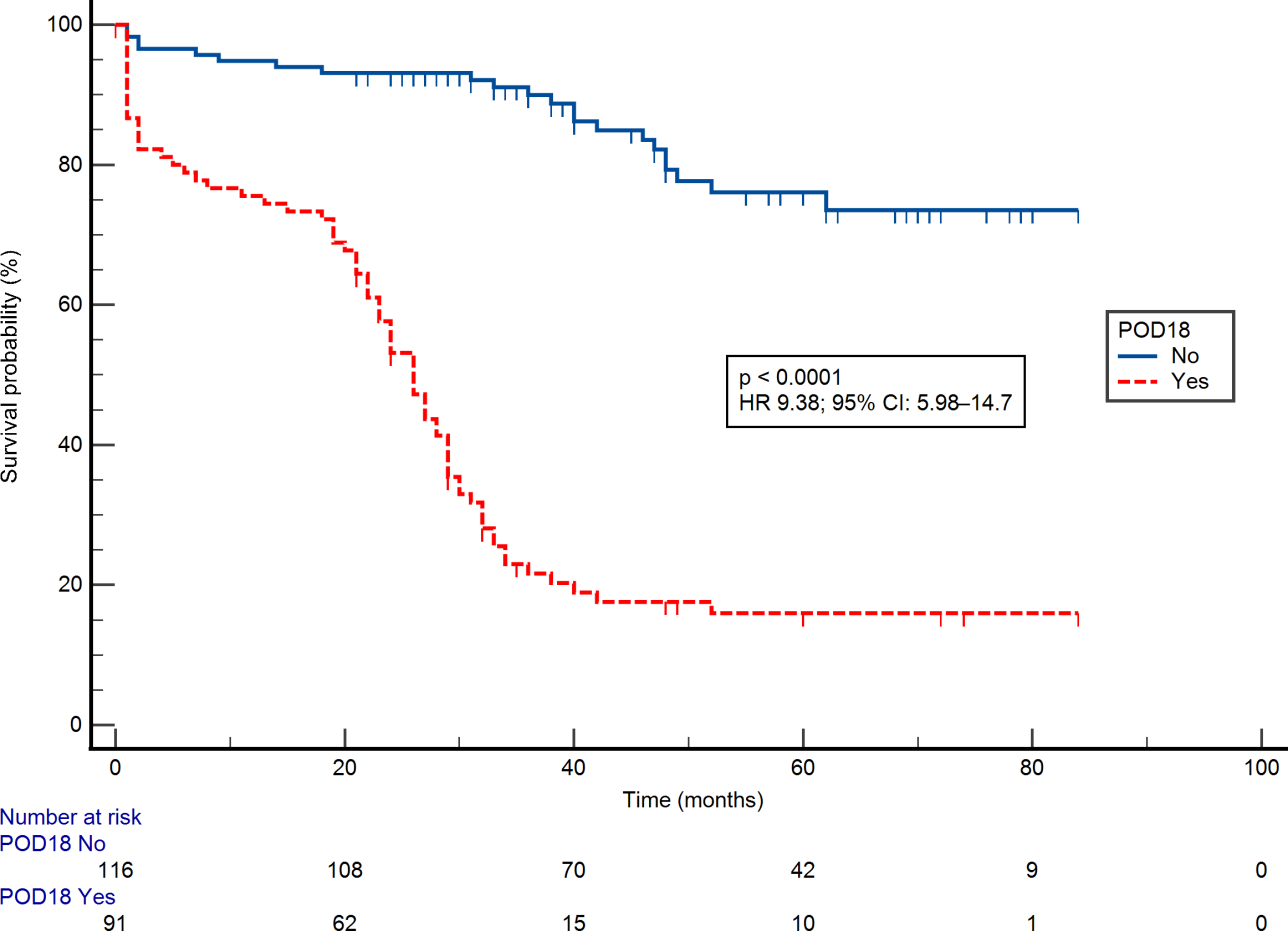
Kaplan–Meier estimates of overall survival according to progression of disease within 18 months (POD18).

**Figure 2:**
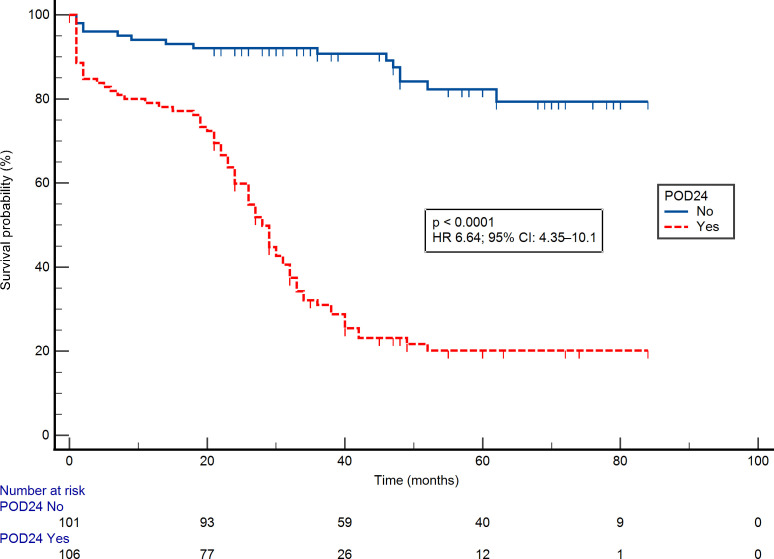
Kaplan–Meier estimates of overall survival according to progression of disease within 24 months (POD24.).

Importantly, POD18 demonstrated superior prognostic discrimination compared with POD24 (C-index 0.742 vs. 0.719), supporting its role as a more informative early dynamic risk marker (**Figures 1**, **2**).

### 3.8 Multivariable analyses

In multivariable Cox regression analysis for overall survival, independent predictors included POD18, comorbidity burden (CCI), impaired performance status (ECOG PS > 2), ISS stage, and lactate dehydrogenase (LDH), as shown in **Table 6**. POD18 showed the strongest association with survival, with a substantially higher hazard ratio (HR 7.15; 95% CI: 4.28–11.96; p < 0.0001) compared with other covariates in the model. The model demonstrated good discriminative ability (C-index 0.830; 95% CI: 0.790–0.870).

**Table 6:** Multivariable Cox proportional hazards regression analysis for overall survival.

Covariate	β coefficient	SE	HR	95%CI	p- value
CCI ≤ 3	-1.1289	0.5737	0.3234	0.1050 to 0.9956	0.049
ECOG PS>2	0.4575	0.2254	1.5802	1.0159 to 2.4578	0.042
POD18	1.9677	0.2621	7.1544	4.2800 to 11.9592	<0.0001
ISS1	-1.2337	0.5105	0.2912	0.1071 to 0.7920	0.016
LDH	0.0007184	0.0003256	1.0007	1.0001 to 1.0014	0.027

Only variables remaining significant in the final multivariable model are shown.

CCI, Charlson Comorbidity Index; ECOG PS, Eastern Cooperative Oncology Group performance status; POD18, progression of disease within 18 months; ISS, International Staging System; LDH, lactate dehydrogenase; SE, Standard error; HR, hazard ratio; CI, confidence interval.

For progression-free survival (PFS), the proportional hazards assumption was violated for ISS and beta-2 microglobulin; therefore, extended Cox regression models incorporating time-dependent covariates were applied.

In this model, ISS, CCI, and haemoglobin remained independently associated with PFS (**Table 7**). ISS was associated with a significantly increased baseline risk of progression (HR 7.26; 95% CI: 2.39–22.02; p < 0.001). However, the time-dependent ISS term was inversely associated with risk (HR 0.55; 95% CI: 0.38–0.80; p = 0.002), indicating that the prognostic impact of ISS decreases over time. The model showed moderate discriminative ability (C-index 0.70; 95% CI: 0.66–0.74).

**Table 7:** Multivariable Cox proportional hazards regression analysis for progression-free survival.

Covariate	β coefficient	SE	HR	95% CI	p-value
CCI > 3	0.6729808	0.2734301	1.9600711	1.1469 to 3.3498	0.01385
Haemoglobin	-0.0169292	0.0052658	0.9832133	0.9731 to 0.9934	0.0013
ISS	1.982808	0.565986	7.2631089	2.3953 to 22.0237	0.00046
β2-microglobulin	0.0292943	0.0268887	1.0297276	0.9769 to 1.0855	0.27595
tt(ISS)	-0.592622	0.190818	0.5528758	0.3804 to 0.8036	0.0019
tt(β2-microglobulin)	-0.0193065	0.0126091	0.9808787	0.9569 to 1.0054	0.12573

Variables remaining significant in the final multivariable model are shown (exception is β2-microglobulin which violated the proportional hazards assumption).

The coefficients for tt(ISS) and tt(β2-microglobulin) represent time-dependent effects, indicating changes in the hazard ratio over time rather than independent baseline effects.

CCI, Charlson Comorbidity Index; ISS, International Staging System; tt, time transformation; SE, Standard error; HR, hazard ratio; CI, confidence interval.

Logistic regression analysis identified ISS stage as the only independent predictor of early mortality within six months (OR 2.37; 95% CI: 1.29–4.35; p = 0.002), indicating that baseline disease burden is the primary determinant of early outcomes.

## 4 Discussion

In this real-world cohort of patients with newly diagnosed multiple myeloma, we demonstrate that early disease progression—particularly within 18 months (POD18)—is a dominant predictor of overall survival and provides stronger prognostic discrimination than the commonly used POD24 threshold. Importantly, our findings extend existing evidence by integrating dynamic disease kinetics with baseline disease burden and patient-related vulnerability, offering a clinically relevant framework for risk stratification in routine practice.

While the adverse prognostic impact of early progression has been consistently reported, our results highlight the incremental value of POD18 as a more sensitive indicator of aggressive disease behaviour compared with POD24. The superior discriminative performance of POD18 suggests that earlier identification of high-risk patients may be achievable, which is of particular relevance in clinical settings where timely risk-adapted interventions are critical. In contrast to prior studies largely based on selected clinical trial populations, our cohort reflects real-world clinical complexity, including advanced disease at presentation and a high burden of comorbidities (13, 15, 16).

A key strength of our analysis lies in the integration of disease-related and patient-related factors. In addition to POD18, comorbidity burden and functional status independently influenced survival, underscoring the multifactorial nature of risk in multiple myeloma. These findings support the concept that outcomes are not determined solely by tumour biology, but also by host-related vulnerability, which is often underrepresented in conventional risk models. In this context, the Charlson Comorbidity Index provides complementary prognostic information and may enhance risk stratification when combined with dynamic markers such as POD18 (13, 16).

Our results also provide insight into the temporal dynamics of prognostic factors. The observed time-dependent effect of ISS on progression-free survival indicates that baseline disease stage has the greatest impact in the early disease phase, while its prognostic relevance diminishes over time. This finding highlights the limitations of static baseline models and supports the need for dynamic, time-updated risk assessment approaches in multiple myeloma. Taken together, these findings suggest that baseline disease burden (ISS stage) influences outcomes through two complementary pathways: early mortality in the initial disease phase and early disease progression (POD18) among patients who survive initial treatment. These observations support a shift from static to dynamic, phase-specific risk assessment across the disease course (15, 16).

Early mortality represents an additional critical dimension of risk. In our cohort, nearly one-fifth of patients died within six months of diagnosis, with advanced ISS stage emerging as the only independent predictor. The causes of early death were predominantly related to end-organ damage and systemic complications, including renal failure, infections, and cardiovascular events, reflecting both high tumour burden and limited physiological reserve. These findings are consistent with prior real-world studies and further emphasise the importance of early disease control and supportive care in this vulnerable patient population (10, 11, 14, 17–20).

The high prevalence of CRAB and SLiM features at diagnosis in our cohort indicates a substantial burden of advanced and symptomatic disease. In this setting, these criteria not only serve as diagnostic markers but also reflect the extent of tumour burden and organ damage, which likely contribute to both early mortality and inferior long-term outcomes (1, 2).

Our analysis should be interpreted in the context of several limitations. First, the retrospective, single-centre design may limit generalisability and introduces the potential for selection bias. Second, cytogenetic data were unavailable or incomplete in a substantial proportion of patients, precluding a comprehensive assessment of biologically defined high-risk disease. As a result, the observed prognostic impact of POD18 may, in part, reflect underlying high-risk cytogenetic features that were not captured. In this context, POD18 should be interpreted as a pragmatic, outcome-based marker of aggressive disease behaviour rather than a direct surrogate of specific biological risk categories (7–9). Third, the relatively small number of patients undergoing ASCT limits the strength of conclusions regarding post-transplant relapse patterns, and these findings should be considered exploratory (21, 22.). Finally, imaging of bone disease was based on conventional radiography, which may underestimate the true extent of skeletal involvement.

Despite these limitations, our study provides clinically relevant insights applicable to routine practice. POD18 represents a simple and accessible dynamic risk marker that does not rely on advanced molecular diagnostics and may therefore be particularly valuable in settings with limited resources. Identification of patients with early progression may support closer monitoring, timely treatment adaptation, and consideration of clinical trial enrolment (15, 16).

In conclusion, early disease progression within 18 months emerges as a robust and clinically meaningful predictor of survival in multiple myeloma, outperforming POD24 in a real-world setting. Our findings support a multidimensional approach to risk stratification that integrates baseline disease burden, dynamic disease kinetics, and patient-related vulnerability. Such an approach may provide a more accurate and actionable framework for personalised management in routine clinical practice.

## Data Availability

The original contributions presented in the study are included in the article/supplementary material. Further inquiries can be directed to the corresponding authors.
